# Corticotropin-releasing hormone as a candidate biomarker for parkinsonian disorders

**DOI:** 10.1093/braincomms/fcae414

**Published:** 2024-11-19

**Authors:** Bárbara Fernandes Gomes, Atul Kumar, Nicholas J Ashton, Sara Hall, Erik Stomrud, Ruben Smith, Henrik Zetterberg, Kaj Blennow, Niklas Mattsson-Carlgren, Oskar Hansson

**Affiliations:** Department of Psychiatry and Neurochemistry, Institute of Neuroscience and Physiology, The Sahlgrenska Academy at the University of Gothenburg, 43180 Mölndal, Sweden; Department of Clinical Sciences Malmö, Clinical Memory Research Unit, Lund University, 20213 Malmö, Sweden; Department of Psychiatry and Neurochemistry, Institute of Neuroscience and Physiology, The Sahlgrenska Academy at the University of Gothenburg, 43180 Mölndal, Sweden; Banner Alzheimer's Institute and University of Arizona, Phoenix, AZ 85006, USA; Banner Sun Health Research Institute, Sun City, AZ 85351, USA; Department of Clinical Sciences Malmö, Clinical Memory Research Unit, Lund University, 20213 Malmö, Sweden; Memory Clinic, Skåne University Hospital, 20502 Malmö, Sweden; Department of Clinical Sciences Malmö, Clinical Memory Research Unit, Lund University, 20213 Malmö, Sweden; Memory Clinic, Skåne University Hospital, 20502 Malmö, Sweden; Department of Clinical Sciences Malmö, Clinical Memory Research Unit, Lund University, 20213 Malmö, Sweden; Memory Clinic, Skåne University Hospital, 20502 Malmö, Sweden; Department of Psychiatry and Neurochemistry, Institute of Neuroscience and Physiology, The Sahlgrenska Academy at the University of Gothenburg, 43180 Mölndal, Sweden; Clinical Neurochemistry Laboratory, Sahlgrenska University Hospital, 43139 Mölndal, Sweden; Department of Neurodegenerative Disease, UCL Institute of Neurology, Queen Square, WC1N 3BG London, UK; UK Dementia Research Institute at UCL, W1T 7NF London, UK; Hong Kong Center for Neurodegenerative Diseases, Clear Water Bay, HKG Hong Kong, China; Wisconsin Alzheimer’s Disease Research Center, University of Wisconsin School of Medicine and Public Health, University of Wisconsin-Madison, Madison, WI 53792, USA; Department of Psychiatry and Neurochemistry, Institute of Neuroscience and Physiology, The Sahlgrenska Academy at the University of Gothenburg, 43180 Mölndal, Sweden; Clinical Neurochemistry Laboratory, Sahlgrenska University Hospital, 43139 Mölndal, Sweden; Paris Brain Institute, ICM, Pitié-Salpêtrière Hospital, Sorbonne University, 75013 Paris, France; Division of Life Sciences and Medicine, Department of Neurology, Neurodegenerative Disorder Research Center, Institute on Aging and Brain Disorders, University of Science and Technology of China and First Affiliated Hospital of USTC, 230026 Hefei, P.R. China; Department of Clinical Sciences Malmö, Clinical Memory Research Unit, Lund University, 20213 Malmö, Sweden; Department of Neurology, Skåne University Hospital, 22185 Lund, Sweden; Wallenberg Center for Molecular Medicine, Lund University, 22184 Lund, Sweden; Department of Clinical Sciences Malmö, Clinical Memory Research Unit, Lund University, 20213 Malmö, Sweden; Memory Clinic, Skåne University Hospital, 20502 Malmö, Sweden

**Keywords:** dementia with Lewy bodies, Parkinson’s disease, atypical parkinsonism, synucleinopathy, fluid biomarker

## Abstract

Disease-specific fluid biomarkers are in demand for parkinsonian syndromes (PS). Corticotropin-releasing hormone (CRH) was proposed as a biomarker for Lewy body disease. As such, this project aimed to confirm CRH as a potential biomarker for different PS. CRH and misfolded α-synuclein (αSyn) were measured in CSF. The primary cohort included Lewy body disease patients (i.e. Parkinson’s disease or dementia with Lewy bodies, *n* = 77), atypical PS (*n* = 37) and non-parkinsonian neurodegenerative diseases (*n* = 164), as well as controls (*n* = 354). A replication cohort included Lewy body disease (*n* = 27), atypical PS (*n* = 58) and controls (*n* = 58). CRH was downregulated in αSyn positive Lewy body disease, αSyn positive controls and in all atypical PS compared with αSyn negative controls (*P* = 3.3e−05, *P* = 3.1e−10, *P* = 2.9e−03). CRH was also decreased in αSyn positive Lewy body disease compared with αSyn negative non-PS (*P* = 2e−03) and correlated with cognitive impairment and inflammation in αSyn positive Lewy body disease. We show that CRH is a promising biomarker for Lewy body disease and atypical PS and its association with inflammation and cognitive decline. Reductions in CRH in Lewy body disease and other PS suggest this decrease may relate to dopaminergic degeneration instead of αSyn pathology.

## Teaser

Fernandes Gomes *et al*. report the downregulation of CSF corticotropin-releasing hormone (CRH) in parkinsonian disorders, and associations with cognition, compared with cognitively unimpaired individuals and non-parkinsonian disorders like Alzheimer’s disease, all of which characterized by α-synuclein seed amplification assay. The authors highlight CRH as a potential biomarker for parkinsonian disorders.

## Graphical Abstract

**Graphical Abstract:**
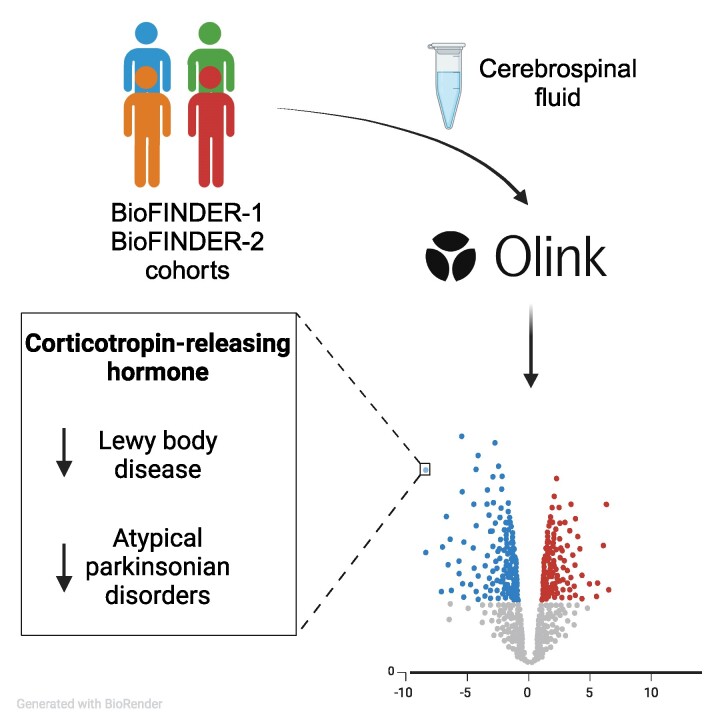

## Introduction

Parkinsonian syndromes (PS) are among the most common neurodegenerative disorders, with Parkinson’s disease affecting over 6 million people worldwide.^1^ Parkinsonian disorders also include dementia with Lewy bodies, which, together with Parkinson’s disease, are commonly denominated as Lewy body diseases (aka neuronal synuclein disease).^2^ Atypical PS include multiple system atrophy (MSA), corticobasal degeneration and progressive supranuclear palsy (PSP), all of which present with various degrees of parkinsonism.^3^ These disorders are mainly diagnosed according to clinical presentations, which often overlap, especially during the early disease stages.^4^ Consequently, the differential diagnosis of PS is challenging, leading to a high rate of misdiagnosis, underscoring the need for disease-specific biomarkers.^5^

Fluid biomarkers have emerged as a practical and low-cost tool to effectively diagnose neurodegenerative diseases.^6^ CSF and blood biomarkers have revolutionized the Alzheimer’s disease field, identifying patients in the early stages of the disease and predicting disease progression.^6,7^ In the Lewy body disease field, recent advances have been made towards the detection of misfolded α-synuclein (αSyn) in CSF using seed amplification assay (SAA), which accurately diagnoses Parkinson’s disease and dementia with Lewy bodies,^8^ and, in some cases, MSA,^9^ distinguishing synucleinopathies from PSP and corticobasal degeneration.^10^ Similarly, plasma neurofilament light chain has shown promise as a high accuracy discriminatory biomarker for atypical PS, increased in MSA, PSP and corticobasal degeneration versus Parkinson’s disease.^11^

Recently, corticotropin-releasing hormone (CRH) has been highlighted as a potential CSF biomarker for Lewy body disease, being decreased in dementia with Lewy bodies compared with Alzheimer’s disease patients and healthy individuals.^12^ CRH is a secretagogue mainly expressed in the paraventricular nucleus (PVN) in the hypothalamus, activating the hypothalamus–pituitary–adrenal (HPA) axis and promoting the release of cortisol by the adrenal cortex, therefore, responsible for stress and inflammatory response.^13^ Brain and CSF studies have implicated CRH in several neurodegenerative diseases. It has been shown to be decreased in the neocortex and CSF of Alzheimer’s disease patients,^14,15^ and in the neocortex of dementia with Lewy bodies,^16^ Parkinson’s disease,^17^ and PSP patients.^17^ Despite this, the mechanism behind the association between CRH and these diseases remains unclear.

We aimed to confirm the link of CRH in Lewy body disease and elucidate its role as a biomarker in the disease, particularly its association with cognition, motor symptoms, psychiatric symptoms and CSF inflammatory markers. We also set out to investigate and compare CRH levels in other diseases such as Alzheimer’s disease, frontotemporal dementia, vascular dementia and atypical PS.

## Materials and methods

The Olink Explore 3072 (Olink Proteomics) was used to analyse the CSF proteome profile of two independent Swedish cohorts using proximity extension assays (Supplementary Fig. 1). SAA was performed to assess αSyn aggregation (SAA− versus SAA+). The pilot cohort Biomarkers for Identifying Neurodegenerative Disorders Early and Reliably 2 (BioFINDER-2) included 632 participants, consisting of 317 SAA− cognitively unimpaired individuals (CUI), 37 SAA+ CUI, 77 patients with SAA+ Lewy body disease (Parkinson’s disease = 46; dementia with Lewy bodies = 31), 37 (SAA− = 35; SAA+ = 2) with atypical PS [PSP = 25 (SAA− = 23; SAA+ = 2); MSA = 12 (SAA− = 12; SAA+ = 0)] and 164 with non-parkinsonian neurodegenerative disorders (SAA− = 120, SAA+ = 44) (Supplementary Table 1). A replication cohort (BioFINDER-1) included 143 individuals, consisting of 58 SAA− CUI, 27 individuals with SAA+ Lewy body disease (Parkinson’s disease = 25; Parkinson’s disease dementia = 1; dementia with Lewy bodies = 1) and 58 with atypical PS [PSP = 28 (SAA− = 26; SAA+ = 1; NA = 1) MSA = 30 (SAA− = 20; SAA+ = 6; NA = 4)] (Supplementary Table 2). CSF CRH was compared between groups and correlated with motor, cognitive, psychiatric and CSF inflammation measures in both cohorts. See Supplementary Materials for details.

### Statistical analysis

Statistical analysis was performed in R (version 4.3.0) and included differential expression analysis, correlation analyses and receiver operating characteristic curve analysis. Statistical significance was set at *P* < 0.05. See Supplementary Materials for details.

## Results

### CRH is downregulated in Lewy body disease and atypical PS

**Figure 1:**
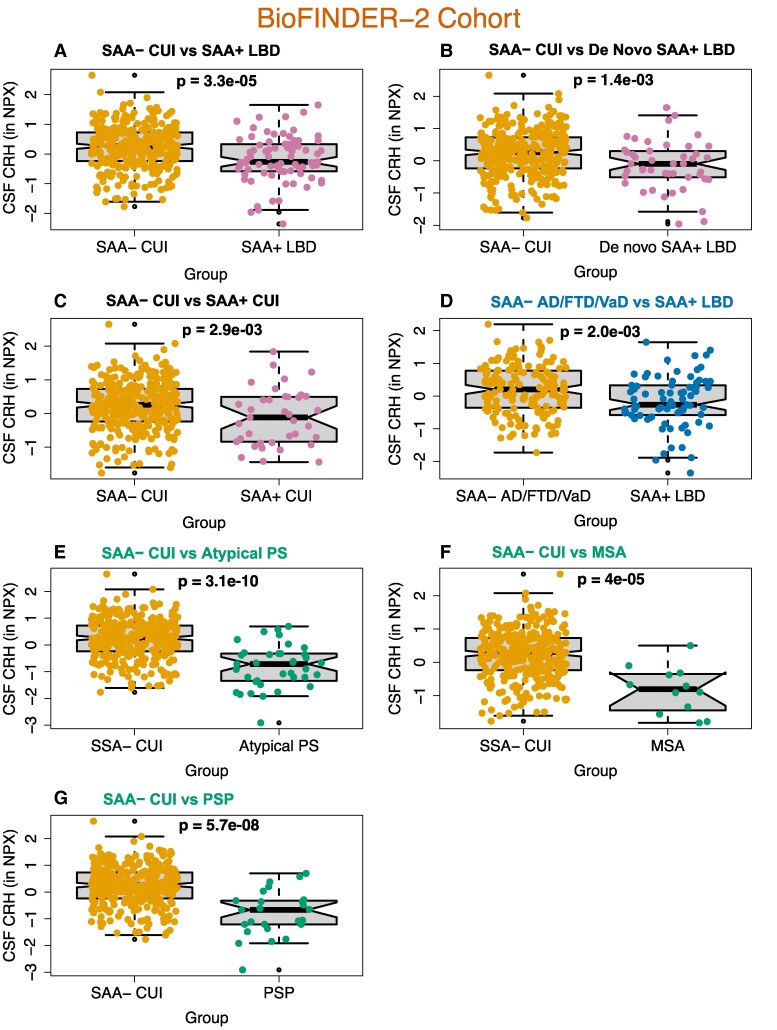
**CSF CRH levels in the BioFINDER-2 cohort**. Comparisons between (**A**) SAA− CUI (*n* = 317) and SAA+ Lewy body disease (*n* = 77), (**B**) SAA− CUI (*n* = 317) and de novo SAA+ Lewy body disease (*n* = 45), (**C**) SAA− CUI (*n* = 317) and SAA+ CUI (*n* = 37), (**D**) SAA− Alzheimer’s disease/frontotemporal dementia/vascular dementia (*n* = 164) and SAA+ Lewy body disease (*n* = 77) and (**E**) SAA− CUI and atypical PS [PSP = 25 (SAA− = 23; SAA+ = 2); MSA = 12 (SAA− = 12; SAA+ = 0)]. Additionally, the two atypical PS subgroups [PSP = 25 (SAA− = 23; SAA+ = 2); MSA = 12 (SAA− = 12; SAA+ = 0)] were compared with SAA− CUI in (**F**) and (**G**). Boxplots show the distribution of data points correspondent to individual patients, in which the mean difference between groups was evaluated by linear models adjusted for age and sex. NPX refers to Olink’s arbitrary unit for relative protein quantification. NPX, normalized protein expression; FTD, frontotemporal dementia; VaD, vascular dementia.

In BioFINDER-2, CSF CRH was reduced in SAA+ Lewy body disease and SAA+ CUI individuals compared with SAA− CUI (*P* = 3.3e−05, AUC = 0.79; *P* = 2.9e−03, AUC = 0.80) (Fig. 1A and C; Supplementary Table 3). CRH was decreased in *de novo* SAA+ Lewy body disease (patients who were not under dopaminergic medication) to a similar extent as in the SAA+ Lewy body disease group (*P =* 1.4e−03, AUC = 0.77) (Fig. 1B; Supplementary Table 3).

CRH was also assessed in non-Lewy body disease, including Alzheimer’s disease/frontotemporal dementia/vascular dementia and atypical PS (Fig. 1D; Supplementary Table 3). Due to the frequent αSyn co-pathology in Alzheimer’s disease, we only compared the SAA− Alzheimer’s disease/frontotemporal dementia/vascular dementia individuals with SAA+ Lewy body disease. We found that CRH was downregulated in SAA+ Lewy body disease compared with SAA− Alzheimer’s disease/frontotemporal dementia/vascular dementia (*P* = 2.0e−03, AUC = 0.69). When comparing SAA− to SAA+ Alzheimer’s disease/frontotemporal dementia/vascular dementia, we found no difference in CRH levels (*P* = 0.2, data not shown). Notably, atypical PS individuals [PSP = 25 (SAA− = 23; SAA+ = 2); MSA = 12 (SAA− = 12; SAA+ = 0)] showed a stronger downregulation in CRH (Fig. 1E) than the Lewy body disease group, when compared with SAA− CUI (*P* = 3.1e−10, AUC = 0.85) (Supplementary Table 3). To ensure CRH downregulation in atypical PS was not influenced exclusively by MSA or PSP, separate comparisons were performed. We found that CRH was decreased in both PSP and MSA when compared with SAA− CUI (Fig. 1F and G). Exclusion of the two SAA+ atypical PS individuals did not affect the results.

### CRH correlates with impaired cognition and inflammation in Lewy body disease

**Figure 2:**
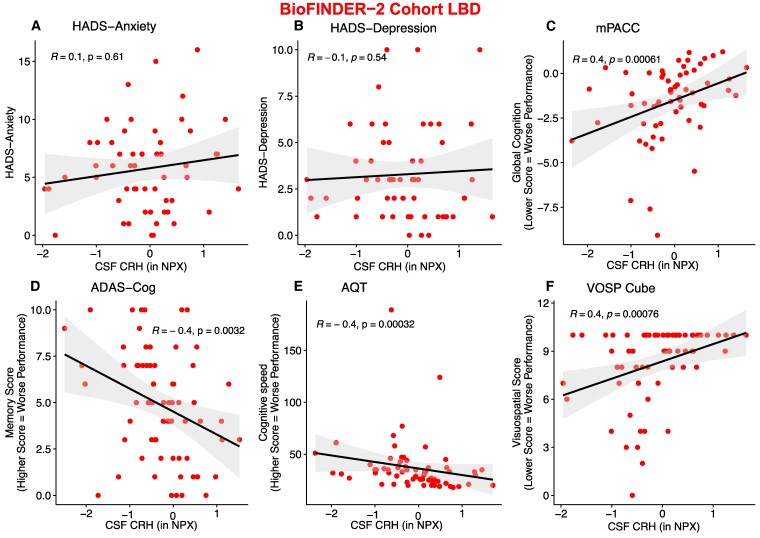
**Correlation of cognition and psychiatric evaluations of Lewy body disease patients and their respective CSF CRH levels in the BioFINDER-2 cohort**. CRH correlation with psychiatric measures (**A**) HADS-Anxiety (*n* = 55) and (**B**) HADS-Depression (*n* = 55). CRH correlation with cognitive measures (**C**) mPACC (*n* = 60), (**D**) ADAS-Cog (*n* = 67), (**E**) AQT (*n* = 67) and (**F**) VOSP Cube (*n* = 65) in the Lewy body disease group. Partial Pearson correlations between variables were performed. NPX refers to Olink’s arbitrary unit for relative protein quantification. *R* represents the correlation coefficient. Individual data represent single patients. NPX, normalized protein expression; HADS, Hospital Anxiety and Depression Scale; mPACC, modified Pre-clinical Alzheimer Cognitive Composite; ADAS-Cog, Alzheimer’s Disease Assessment Scale–Cognitive Subscale; AQT, A Quick Test of Cognitive Speed; VOSP, Visual Object and Space Perception Battery.

To elucidate what clinical impact CRH may have in Lewy body disease, we assessed its relationship with psychiatric and cognitive measures (Fig. 2). Since CRH is associated with depression and anxiety disorders,^18,19^ we performed correlations between anxiety and depression measures and CRH (Fig. 2A and B).

We found no correlation between anxiety or depression and CRH in Lewy body disease. However, CRH downregulation correlated with impaired cognition in Lewy body disease individuals (Fig. 2C–F), specifically, worse global cognition (mPACC, *R* = 0.4, *P* = 0.00061), memory score (ADAS-Cog, *R* = −0.4, *P* = 0.0032), cognitive speed (AQT, *R* = −0.4, *P* = 0.00032) and visuospatial score (VOSP, *R* = 0.4, *P* = 0.00076).

**Figure 3:**
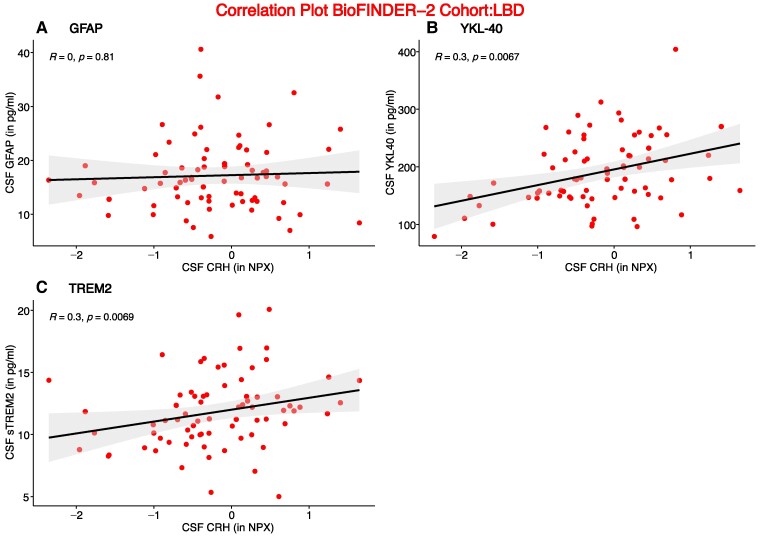
**Inflammation markers association with CRH in Lewy body disease patients**. Correlation of (**A**) GFAP and CRH, (**B**) YKL-40 and CRH and (**C**) TREM2 and CRH, in Lewy body disease patients (*n* = 67). Partial Pearson correlations between variables were performed. NPX refers to Olink’s arbitrary unit for relative protein quantification. *R* represents the correlation coefficient. Individual data represent single patients. NPX, normalized protein expression.

As CRH is involved in the inflammatory response,^13^ correlations between CRH and inflammatory markers glial fibrillary acidic protein (GFAP), chitinase-3-like protein 1 (YKL-40) and soluble triggering receptor expressed on myeloid cells 2 (sTREM2) were assessed (Fig. 3). We observed weak but significant correlations between CRH and YKL-40 (*R* = 0.3, *P* = 0.0067) and sTREM2 (*R* = 0.3, *P* = 0.0069) in the Lewy body disease group, but no correlation with GFAP.

Motor symptoms and CRH in Lewy body disease were compared, but no significant correlations were found. Similarly, a link between CRH and psychiatric, motor and cognitive measurements, as well as inflammatory markers, was investigated in atypical PS, but no association was observed (Supplementary Figs. 2 and 3).

### Replication cohort

**Figure 4:**
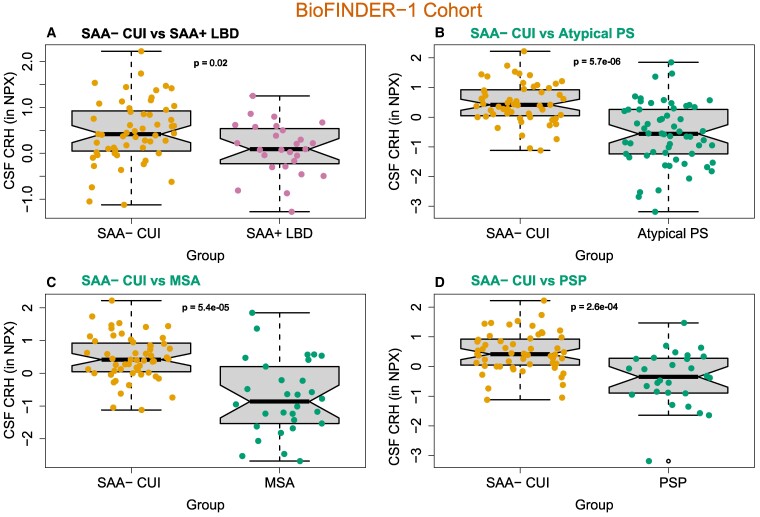
**CSF CRH levels in the BioFINDER-1 cohort**. Comparisons between (**A**) SAA− CUI (*n* = 58) and SAA+ Lewy body disease (*n* = 27), (**B**) SAA− CUI (*n* = 58) and atypical PS (*n* = 58), (**C**) SAA− CUI and MSA (*n* = 30) and (**D**) SAA− CUI and PSP (*n* = 28). Boxplots show the distribution of data points correspondent to individual patients, in which the mean difference between groups was evaluated using linear models adjusted for age and sex. NPX refers to Olink’s arbitrary unit for relative protein quantification. NPX, normalized protein expression.

To ensure our findings were comprehensive and reflective of the studied population, CSF CRH was analysed in the independent cohort BioFINDER-1 (Fig. 4). CRH was decreased in SAA+ Lewy body disease compared with SAA− CUI (*P* = 0.02, AUC = 0.65) (Fig. 4A; Supplementary Table 3). Regarding atypical PS, CRH was reduced when compared with SAA− CUI (*P* = 5.7e−06, AUC = 0.78) (Fig. 4B; Supplementary Table 3), even when splitting atypical PS by diagnosis (Fig. 4C and D). The relationship between CRH and cognition (ADAS-Cog and AQT) was assessed in all groups in BioFINDER-1, but no correlation was found.

A summary of the main findings can be found in Supplementary Table 4.

## Discussion

There is an urgent demand for novel robust and disease-specific biomarkers in the parkinsonian disease field, both to improve patient management in clinical practice and to study disease mechanisms *in vivo*. We confirm CRH as a candidate biomarker for Lewy body disease and show novel data for its utility in atypical PS. We also discuss the potential mechanism driving CSF CRH levels and its biological implications.

Our findings suggest that CRH is downregulated in SAA+ Lewy body disease and atypical PS individuals, compared with SAA− CUI and SAA− Alzheimer’s disease/frontotemporal dementia/vascular dementia. Furthermore, we observed that dopaminergic medication had no impact on the levels of CRH, as seen in the *de novo* SAA+ Lewy body disease group. CRH levels were also reduced in SAA+ CUI (pre-clinical Lewy body disease), in which the presence of αSyn could indicate a higher risk for disease progression.^20^ This suggests CRH dysregulation might occur early in the disease course, despite the limited pre-clinical sample size. Additionally, CRH was decreased in SAA+ Lewy body disease compared with SAA− Alzheimer’s disease/frontotemporal dementia/vascular dementia (with an overlap between groups). These data are corroborated by a recent study, also based on proximity extension assay, which identified CRH as a potential biomarker, discriminating dementia with Lewy bodies from Alzheimer’s disease.^12^

We initially hypothesized the decrease in CRH levels was due to αSyn pathology, as seen when comparing SAA+ and SAA− CUI. However, CRH was the most downregulated in atypical PS, a group consisting of PSP and MSA. When individually comparing these two groups with SAA− CUI, we observed that MSA did not drive the decreased levels of CRH in atypical PS. Despite both PSP and MSA presenting with parkinsonism, their pathophysiology is vastly different. MSA has a pathological aggregation of misfolded αSyn in glial cytoplasmatic inclusions. In contrast, in PSP, the main feature is the presence of neurofibrillary tangles predominantly composed of hyperphosphorylated tau, compromising considerably different brain structures.^21^ Hence, as CRH is decreased to a similar extent in PSP and MSA, and it was not altered when comparing SAA− to SAA+ Alzheimer’s disease/frontotemporal dementia/vascular dementia, an αSyn-independent mechanism might be behind the decrease of CRH levels in these diseases. Since this downregulation seems to be only partly affected by the presence of αSyn, we propose the decrease of CRH levels in Lewy body disease and atypical PS could be related to dopaminergic dysfunction instead. The denervation of dopaminergic neurons is common in Lewy body disease and atypical PS,^22,23^ and the PVN, where CRH is mainly produced, is subject to excitatory and inhibitory dopaminergic regulation.^24-26^ As such, the loss of dopaminergic innervation occurring in these diseases might contribute to CRH dysregulation.

The decrease of CRH in Lewy body disease and atypical PS carries several biological implications, as CRH is involved in stress, inflammation, metabolism and reproduction, among others.^27^ Consequently, abnormal levels of CRH would impact factors known to contribute to neurodegenerative diseases, particularly inflammation.^28^ CRH dysregulation would suggest a disproportional inflammatory response, which is commonly observed in these diseases. Indeed, this link is strengthened by the correlation we observed between CRH and two neuroinflammation markers, YKL-40 and sTREM2. Similarly, the association between CRH and cognitive impairment observed here suggests that fluctuations in CRH have a significant impact on cognition, as reported in previous studies.^29,30^ Importantly, despite no correlation with depression and anxiety rating scales, changes in CRH levels may imply a dysregulation of the HPA axis, indicating an inappropriate response to stress, potentially involving neurodegenerative processes.

This study is not without limitations. The absence of longitudinal clinical data prevented us from assessing if pre-symptomatic SAA+ would progress to a symptomatic parkinsonian disorder, as well as how CRH levels change over the course of the disease. Furthermore, these samples were analysed on a multiplex platform, which is unsuitable for application in clinical routine. Therefore, future work should focus on developing a single-plex assay targeting CRH, such as an immuno- or mass spectrometric assay. Finally, some of the subgroup analyses suffered from low sample numbers, meaning replication studies are needed.

## Conclusion

We highlight CRH as a possible biomarker for parkinsonian disorders. Furthermore, we show the potential of CRH as a novel biomarker for atypical parkinsonian disorders, namely MSA and PSP. We propose that, given that CRH levels are decreased in Lewy body disease patients and SAA+ CUI, in comparison to SAA− CUI, αSyn might be responsible for the decrease in CRH levels. However, because this decrease is not limited to synucleinopathies and it does not occur when comparing SAA− to SAA+ Alzheimer’s disease/frontotemporal dementia/vascular dementia, dopaminergic dysfunction may instead be the cause of CRH decrease, which in turn could be associated to the stress and inflammatory aspect of these diseases. These data encourage targeted studies to measure CRH in CSF and further evaluate its potential as a biomarker to strengthen the robustness of our findings and to understand what mechanism CRH might reflect in parkinsonian disorders.

## Supplementary Material

fcae414_Supplementary_Data

## Data Availability

Pseudonymized data will be shared by request from qualified academic investigators for the sole purpose of replicating procedures and results presented in the article and as long as data transfer is in agreement with EU legislation on the general data protection regulation and decisions by the Swedish Ethical Review Authority and Region Skåne, which should be regulated in a material transfer agreement. Code generated is available at https://github.com/atulkumar1301/PD_CRH.git.
