# Supplementary material for: Corticotropin-releasing hormone as a candidate biomarker for parkinsonian disorders
Source: Brain Commun. 2024 Nov 19;6(6):fcae414. doi: 10.1093/braincomms/fcae414 (PMC11601160; doi:10.1093/braincomms/fcae414)
Supplement: fcae414_Supplementary_Data [file fcae414_supplementary_data.pdf]

Supplementary material

Materials and Methods

Participants

We included 635 participants from the Swedish BioFINDER-2 cohort (NCT03174938),[1] a study focused on discovering and creating biomarkers for diagnosing neurodegenerative disorders. All participants were recruited at the Skåne University Hospital, Sweden, between 2017 and 2020. The participants consisted of 317 controls, 37 SAA+ cognitively unimpaired individuals (CUI), 77 patients with LBD, 37 patients with atypical PS, and 164 patients with other non-parkinsonian neurodegenerative disorders. All participants underwent lumbar puncture and clinical examinations. The LBD group comprised 46 patients with PD (25 were drug naïve or *de novo*) and 31 with DLB (20 were drug naïve or *de novo*). The atypical parkinsonian group consisted of 25 patients with PSP and 12 with MSA. Finally, the group with other neurodegenerative disorders consisted of 128 patients with AD, 17 with FTD and 19 with VaD. Participants' motor function was evaluated using the Unified Parkinson's Disease Rating Scale (UPDRS) - part III.[2] The modified Pre-clinical Alzheimer Cognitive Composite (mPACC)[3] evaluated global cognition, which included tasks that measure memory, executive function, attention, and language abilities. In addition, we evaluated more precise cognitive areas, such as cognitive speed, using the AQT (A Quick Test of cognitive speed and attention)[4,5] and memory function (delayed memory recall from ADAS-Cog).[6] The individual's visuospatial function was assessed using the visual object and space perception battery (VOSP), specifically the cube analysis subtest.[7] To measure the levels of anxiety and depression, the self-rated Hospital Anxiety and Depression Scale (HADS) assessed levels of depression (HADS-D) and anxiety (HADS-A). HADS has superior psychometric qualities (range, 0–21).[8]

To validate our results in an independent set of participants, we assessed 143 individuals from the Swedish BioFINDER-1 study (NCT01208675)[9] who were recruited between 2007 and 2015. CSF samples were analysed from 58 CUI, 27 individuals with LBD (25 with PD, one with PD with dementia (PDD), and one with DLB), and 58 individuals with atypical PS (28 with PSP and 30 with MSA).

In both BioFINDER-2 and BioFINDER-1, the CUI group included both cognitively healthy individuals and participants with subjective cognitive decline (SCD) who demonstrated normal performance on a comprehensive cognitive test battery administered by expert neuropsychologists. Participants were required to meet the

following criteria: a) age between 40 and 100 years; b) a minimum MMSE score of 24 points; c) proficiency in the Swedish language. Crucially, none of the control individuals included in the study met the clinical criteria for PD, prodromal dementia with DLB, atypical PS, or any other neurological disease at the baseline. Patients with LBD were required to: a) fulfil the criteria for PD,[10] dementia due to DLB,[11] or PDD,[12] and b) be fluent in Swedish. Patients with atypical parkinsonian disorders were required to: a) meet the consensus statement for MSA,[13] the criteria for PSP,[14] or the diagnosis guidelines for CBS[15]; and b) be fluent in Swedish. Patients with other non-parkinsonian neurodegenerative disorders were required to: a) fulfil the DSM-5 criteria for dementia (major neurocognitive disorder) due to AD, FTD or VaD[16,17]; and b) be fluent in Swedish. Exclusion criteria for all groups were: a) having a significant unstable systemic illness that makes it difficult to participate in the study; b) current significant alcohol or substance misuse; and c) refusing lumbar puncture. All participants gave written informed consent before entering the study. The study procedure was approved by the local ethics committee at Lund University in Sweden (ethical approval reference' Dnr 2016/1053 for BioFINDER-2, Dnr 2010/156 for BioFINDER-1) and conducted according to the Helsinki Declaration.

Lumbar puncture

The lumbar puncture procedure was conducted before noon in the L3/L4 or L4/L5 interspace on participants who were not fasting.[18] The CSF samples were gathered in polypropylene tubes, mixed to prevent variations in concentration, and subjected to centrifugation at a temperature of 4 °C within 30 minutes. The centrifugation was carried out at a force of 2000 g for 10 minutes to eliminate cells and unwanted particles. Subsequently, the samples were divided into aliquots and preserved at -80 °C until biochemical analysis.

CSF analyses

The Olink Explore 3072 platform, as well as the detection of αSyn aggregation by real-time quaking-induced conversion seed amplification assay (SAA) were performed as previously described in Pereira and Kumar *et al.*.[19] All data shown here pertains solely to the proteins included in this study (CRH, GFAP, YKL40, and sTREM2).

Statistics and reproducibility

All statistical analyses were performed in R (version 4.3.0). To identify specific significantly upregulated or downregulated proteins, we performed a differential expression analysis on the protein levels obtained from the Olink platform. This analysis used a generalised linear model with a logit link function and binomial distribution. We also considered age and sex as covariates. Partial correlation analyses evaluated the association between CRH levels and psychiatric, cognitive and inflammation measures. Age and sex were controlled for in these analyses. A second analysis of protein levels using the Olink platform was performed in the same manner in the BioFINDER-1 cohort. We conducted a ROC curve analysis using 5,000 bootstrapped samples to evaluate the capacity of significant proteins to differentiate between various groups, calculating the AUC, accuracy, specificity, and confidence intervals for all group comparisons. Each set of association analyses was corrected for false discovery rate (FDR) using Benjamini and Hochberg procedure.[20] Associations below FDR corrected p-value of 0.05 were considered significant. The biomarker analysis was blinded, but data collection and analysis process lacked randomisation and blinding to the experimental groups.

Results

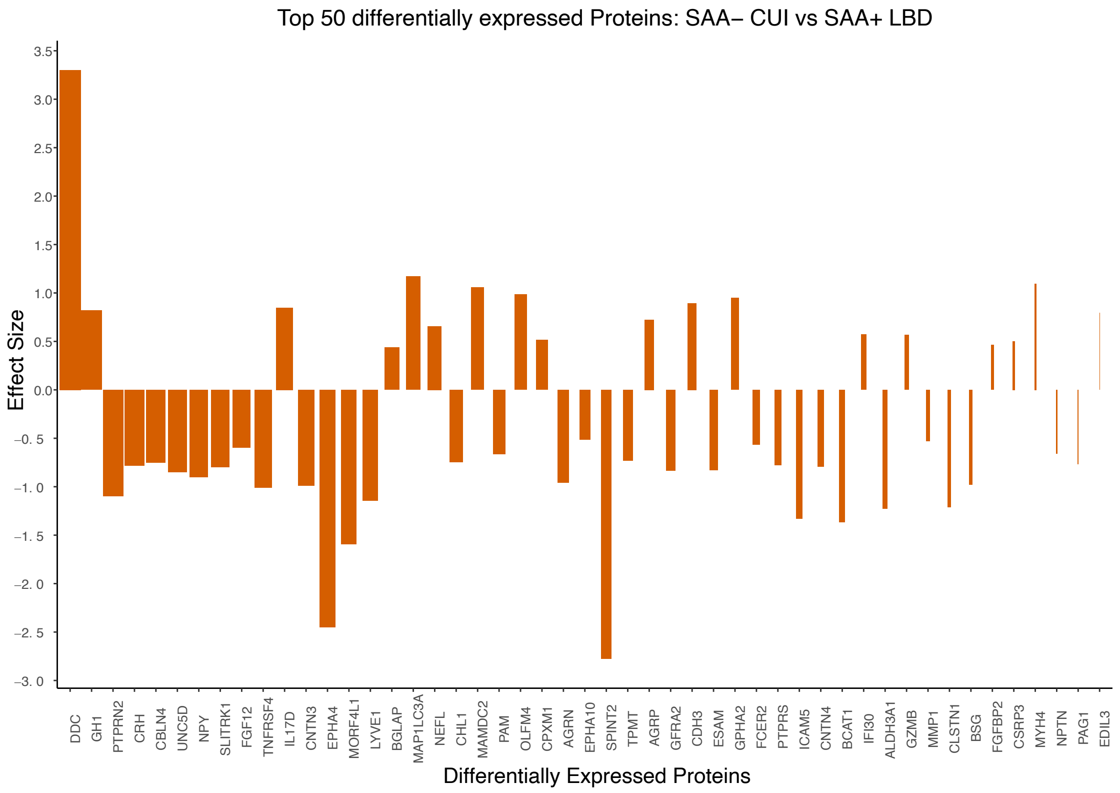

Supplementary Figure 1. Top 50 differentially expressed proteins in SAA- CUI and SAA+ LBD identified in Pereira and Kumar et al..[19] Bars represent fold-change of each protein in SAA+ LBD patients, compared to SAA- CUI individuals. Logistic regression adjusted for age and sex was performed. Width of bars denotes the significance level of the effect size. Proteins were identified with the Olink Explore 3072 panel. Abbreviations: SAA, seed amplification assay; CUI, cognitively unimpaired individuals or controls; LBD, Lewy body disease.

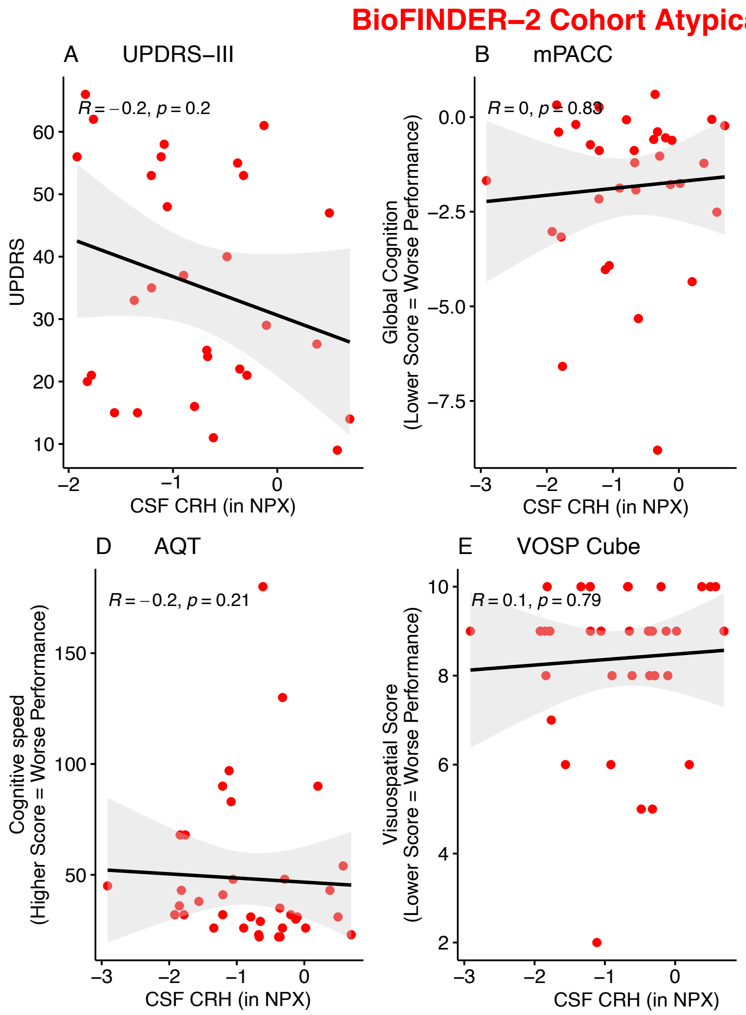

Supplementary Figure 2. Correlation of motor and cognitive evaluations of atypical PS patients and their respective CSF CRH levels in the BioFINDER-2 cohort. CRH correlation with **(A)** Unified Parkinson's Disease Rating Scale (UPDRS-III, n=30) **(B)** mPACC (n=34), **(C)** ADAS-Cog (n=40), **(D)** AQT (n=37), and **(E)** VOSP Cube (n=37), in atypical PS patients. Individual data represents single patients. Partial Pearson correlations between variables were performed. *R* represents the correlation coefficient. NPX refers to Olink's arbitrary unit for relative protein quantification. Abbreviations: BioFINDER, Biomarkers For Identifying Neurodegenerative Disorders Early and Reliably; NPX, normalized protein expression; mPACC, modified Pre-clinical Alzheimer Cognitive Composite; ADAS-Cog, Alzheimer's Disease Assessment Scale–Cognitive Subscale; AQT, A Quick Test of Cognitive Speed; VOSP, Visual Object and Space Perception Battery.

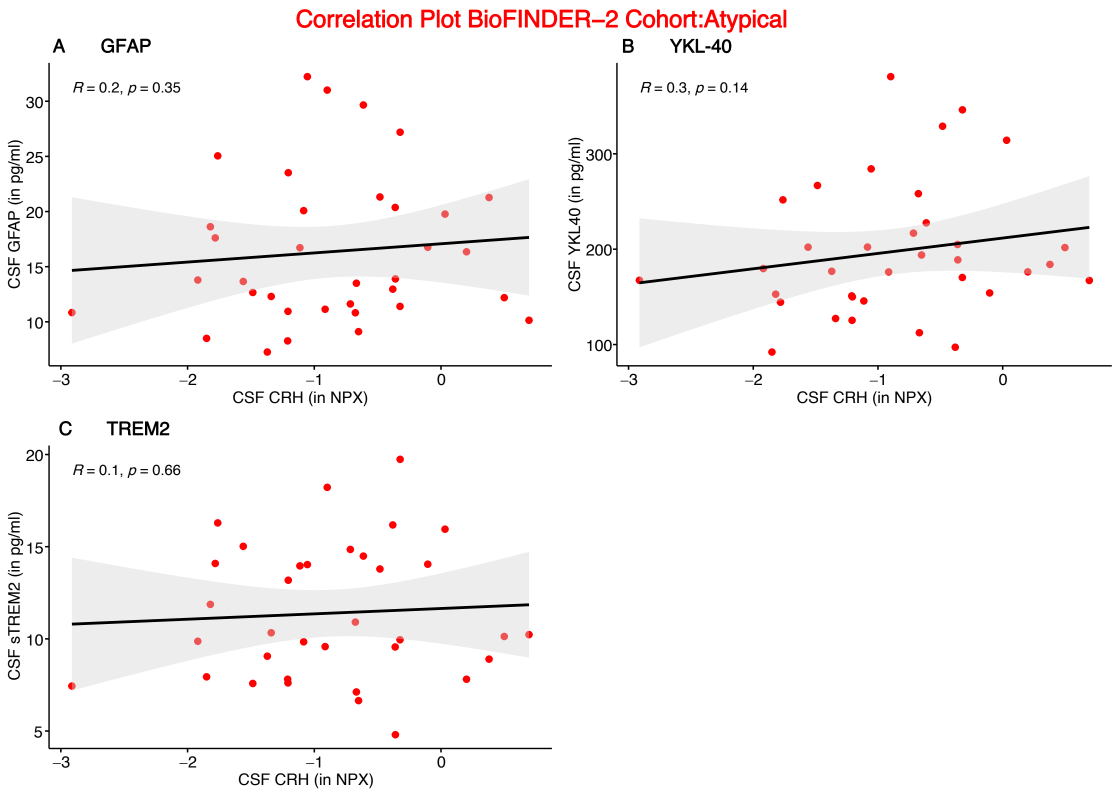

Supplementary Figure 3. Inflammation markers association with CRH in atypical PS patients. Correlation of **(A)** GFAP and CRH, **(B)** YKL-40 and CRH, and **(C)** TREM2 and CRH, in atypical PS patients (n=35). Individual data represents single patients. Partial Pearson correlations between variables were performed. *R* represents the correlation coefficient. NPX refers to Olink's arbitrary unit for relative protein quantification. Abbreviations: BioFINDER, Biomarkers For Identifying Neurodegenerative Disorders Early and Reliably; NPX, normalized protein expression; GFAP, glial fibrillary acidic protein; YKL-40, chitinase-3-like protein 1; sTREM2, soluble triggering receptor expressed on myeloid cells 2.

Supplementary Table 1. Characteristics of the BioFINDER-2 cohort.

	**CUI (n = 354)**	**LBD (n = 77)**	**Atypical PS (n = 37)**	**Non-PS (n = 164)**
**Age (Years)**	63.4 (14.4)	71.1 (9.2)	67.5 (8.4)	72.7 (7.4)
**Sex (M/F)**	168/186	55/22	19/18	80/84
**Education (Years)**	12.8 (3.4)	12.9 (4.0)	12 (23.2)	12.1 (4.0)
**Diagnostic groups**	-	46/31 (PD/DLB)	25/12 (PSP/MSA)	128/17/19 (AD/FTD/VaD)
**αSyn (SAA-/SAA+)**	317/37	77/0	35/2	120/44
**CSF CRH (NPX units on a log2 scale)**	0.19 (0.75)	-0.17 (0.8)	-0.8 (0.8)	0.2 (0.8)

Data are presented as means followed by (standardized deviation), unless stated otherwise.

Abbreviations: BioFINDER, Biomarkers For Identifying Neurodegenerative Disorders Early and Reliably; CUI, cognitively unimpaired individuals; LBD, Lewy Body disease; non-PS, non-Parkinsonian disorders; PS, Parkinsonian syndrome; PD, Parkinson's disease; DLB, dementia with Lewy bodies; PSP, progressive supranuclear palsy; MSA, multiple system atrophy; AD, Alzheimer's disease; FTD, frontotemporal dementia; VaD, vascular dementia; M/F, male/female; CSF, cerebrospinal fluid; CRH, Corticotropin-releasing hormone levels in normalized protein expression (NPX) units on a log2-scale.

Supplementary Table 2. Characteristics of the independent validation cohort (BioFINDER-1) with CSF samples.

	**CUI (n = 58)**	**LBD (n = 27)**	**Atypical PS (n = 58)**
**Age (Years)**	69.7 (8.5)	68.7 (8.8)	68.0 (8.6)
**Sex (M/F)**	25/33	15/12	32/26
**Education (Years)** [a]	13.6 (3.4)	13.6 (4.7)	12.1 (4.4)
**Diagnostic groups**	-	25/1/1 (PD/PDD/DLB)	28/30 (PSP/MSA)
**αSyn (SAA-/SAA+)**	58/0	27/0	46/7*
**CSF CRH (NPX units on a log2 scale)**	0.42 (0.7)	0.1 (0.6)	0.6 (1)

Data are presented as means followed by (standardized deviation), unless stated otherwise.

Abbreviations: BioFINDER, Biomarkers For Identifying Neurodegenerative Disorders Early and Reliably; CUI, cognitively unimpaired individuals; LBD, Lewy Body disease; PS, Parkinsonian syndrome; PD, Parkinson's disease; DLB, dementia with Lewy bodies; PSP, progressive supranuclear palsy; MSA, multiple system atrophy; M/F, male/female; CSF, cerebrospinal fluid; CRH, Corticotropin-releasing hormone levels in normalized protein expression (NPX) units on a log2-scale.

*NA=5

Supplementary Table 3. Receiver operating characteristic curve analysis for the comparison between different groups, based on the best diagnostic threshold.

	**AUC**	**Sensitivity**	**Specificity**	**L95**	**U95**	**Best diagnostic threshold**
	**BioFINDER-2**					
SAA- CUI vs SAA+ LBD	0.79	0.65	0.82	0.73	0.84	0.26
SAA- CUI vs De Novo SAA+ LBD	0.77	0.71	0.71	0.70	0.85	0.12
SAA- CUI vs SAA+ CUI	0.80	0.76	0.74	0.72	0.87	0.11
SAA- AD/FTD/VaD vs SAA+ LBD	0.69	0.64	0.70	0.62	0.76	0.33
SAA- CUI vs Atypical PS	0.85	0.77	0.81	0.79	0.92	0.11
	**BioFINDER-1**					
SAA- CUI vs SAA+ LBD	0.65	0.63	0.62	0.53	0.77	0.32
SAA- CUI vs Atypical PS	0.78	0.64	0.88	0.70	0.87	0.55

Abbreviations: BioFINDER, Biomarkers For Identifying Neurodegenerative Disorders Early and Reliably; CUI, cognitively unimpaired individuals; LBD, Lewy Body disease; PS, Parkinsonian syndrome; AD, Alzheimer's disease; FTD, frontotemporal dementia; VaD, vascular dementia; AUC, area under the curve; L95 and U95, upper and lower 95% confidence interval

Supplementary Table 4. Summary of the main findings.

**Protein levels**	**Effect**	**SE**	***P*-value**	**L95**	**U95**
		**BioFINDER-2**			
SAA- CUI vs SAA+ LBD	-0.78	0.19	3.27E-05	-1.17	-0.42
SAA- CUI vs De novo SAA+ LBD	-0.73	0.23	1.39E-03	-1.20	-0.29
SAA- CUI vs SAA+ CUI	-0.75	0.25	2.87E-03	-1.26	-0.27
SAA- CUI vs Atypical PS	-1.91	0.30	3.13E-10	-2.55	-1.36
SAA- CUI vs MSA	-1.78	0.44	6.02E-05	-2.73	-0.97
SAA- CUI vs PSP	-1.79	0.34	1.15E-07	-2.51	-1.17
SAA- AD/FTD/VaD vs SAA+ LBD	-0.59	0.19	1.99E-03	-0.98	-0.22
		**BioFINDER-1**			
SAA- CUI vs SAA+ LBD	-0.60	0.15	2.03E-02	-0.96	-0.03
SAA- CUI vs Atypical PS	-1.32	0.29	5.68E-06	-1.95	-0.79
**Correlations (BioFINDER-2, LBD)**	***R***	***P*-value**			
HADS-Depression	-0.1	0.54	-	-	-
HADS-Anxiety	0.1	0.61	-	-	-
mPACC	0.4	6.1e-04	-	-	-
ADAS-Cog	-0.4	3.2e-03	-	-	-
AQT	-0.4	3.2e-04	-	-	-
VOSP	0.4	7.6e-04	-	-	-
GFAP	0	0.81	-	-	-
YKL-40	0.3	6.7e-03	-	-	-
sTREM2	0.3	6.9e-03	-	-	-

Abbreviations: BioFINDER, Biomarkers For Identifying Neurodegenerative Disorders Early and Reliably; CUI, cognitively unimpaired individuals; LBD, Lewy Body disease; PS, Parkinsonian syndrome; PSP, progressive supranuclear palsy; MSA, multiple system atrophy; AD, Alzheimer's disease; FTD, frontotemporal dementia; VaD, vascular dementia; SE, standard error; L95 and U95, upper and lower 95% confidence interval; R, Pearson correlation coefficient; HADS, Hospital Anxiety and Depression Scale; mPACC, modified Pre-clinical Alzheimer Cognitive Composite; ADAS-Cog, Alzheimer's Disease Assessment Scale–Cognitive Subscale; AQT, A Quick Test of Cognitive Speed; VOSP, Visual Object and Space Perception Battery; GFAP, glial fibrillary acidic protein; YKL-40, chitinase-3-like protein 1; sTREM2, soluble triggering receptor expressed on myeloid cells 2.

References

1. Palmqvist S, Janelidze S, Quiroz YT, *et al*. Discriminative Accuracy of Plasma Phospho-tau217 for Alzheimer Disease vs Other Neurodegenerative Disorders. *Jama*. Aug 25 2020;324(8):772-781. doi:10.1001/jama.2020.12134
2. Fahn S, Marsden CD, Goldstein M, Calne DB. *Recent Developments in Parkinson's Disease*. Macmillan Healthcare Information; 1987.
3. Papp KV, Rentz DM, Orlovsky I, Sperling RA, Mormino EC. Optimizing the preclinical Alzheimer's cognitive composite with semantic processing: The PACC5. *Alzheimers Dement (N Y)*. Nov 2017;3(4):668-677. doi:10.1016/j.trci.2017.10.004
4. Jalakas M, Palmqvist S, Hall S, *et al*. A quick test of cognitive speed can predict development of dementia in Parkinson's disease. *Sci Rep*. Oct 28 2019;9(1):15417. doi:10.1038/s41598-019-51505-1
5. Abwender DA, Swan JG, Bowerman JT, Connolly SW. Qualitative analysis of verbal fluency output: review and comparison of several scoring methods. *Assessment*. Sep 2001;8(3):323-38. doi:10.1177/107319110100800308
6. Mohs RC. The Alzheimer's Disease Assessment Scale. *International Psychogeriatrics*. 1996;8(2):195-203. doi:10.1017/S1041610296002578
7. Rapport LJ, Millis SR, Bonello PJ. Validation of the Warrington theory of visual processing and the Visual Object and Space Perception Battery. *J Clin Exp Neuropsychol*. Apr 1998;20(2):211-20. doi:10.1076/jcen.20.2.211.1169
8. Djukanovic I, Carlsson J, Årestedt K. Is the Hospital Anxiety and Depression Scale (HADS) a valid measure in a general population 65-80 years old? A psychometric evaluation study. *Health Qual Life Outcomes*. Oct 4 2017;15(1):193. doi:10.1186/s12955-017-0759-9
9. Palmqvist S, Tideman P, Cullen N, *et al*. Prediction of future Alzheimer's disease dementia using plasma phospho-tau combined with other accessible measures. *Nat Med*. Jun 2021;27(6):1034-1042. doi:10.1038/s41591-021-01348-z
10. Gelb DJ, Oliver E, Gilman S. Diagnostic criteria for Parkinson disease. *Arch Neurol*. Jan 1999;56(1):33-9. doi:10.1001/archneur.56.1.33
11. McKeith IG, Dickson DW, Lowe J, *et al*. Diagnosis and management of dementia with Lewy bodies: third report of the DLB Consortium. *Neurology*. Dec 27 2005;65(12):1863-72. doi:10.1212/01.wnl.0000187889.17253.b1
12. Emre M, Aarsland D, Brown R, *et al*. Clinical diagnostic criteria for dementia associated with Parkinson's disease. *Mov Disord*. Sep 15 2007;22(12):1689-707; quiz 1837. doi:10.1002/mds.21507
13. Gilman S, Wenning GK, Low PA, *et al*. Second consensus statement on the diagnosis of multiple system atrophy. *Neurology*. Aug 26 2008;71(9):670-6. doi:10.1212/01.wnl.0000324625.00404.15
14. Höglinger GU, Respondek G, Stamelou M, *et al*. Clinical diagnosis of progressive supranuclear palsy: The movement disorder society criteria. *Mov Disord*. Jun 2017;32(6):853-864. doi:10.1002/mds.26987
15. Armstrong MJ, Litvan I, Lang AE, *et al*. Criteria for the diagnosis of corticobasal degeneration. *Neurology*. Jan 29 2013;80(5):496-503. doi:10.1212/WNL.0b013e31827f0fd1
16. Jack CR, Jr., Bennett DA, Blennow K, *et al*. NIA-AA Research Framework: Toward a biological definition of Alzheimer's disease. *Alzheimers Dement*. Apr 2018;14(4):535-562. doi:10.1016/j.jalz.2018.02.018
17. American Psychiatric Association. *Diagnostic and statistical manual of mental disorders (5th ed.)*.

18. Hu B, Chen TM, Liu B, *et al*. Optimal management after paediatric lumbar puncture: a randomized controlled trial. *BMC Neurol*. Apr 13 2019;19(1):64. doi:10.1186/s12883-019-1275-9
19. Pereira JB, Kumar A, Hall S, *et al*. DOPA decarboxylase is an emerging biomarker for Parkinsonian disorders including preclinical Lewy body disease. *Nat Aging*. Oct 2023;3(10):1201-1209. doi:10.1038/s43587-023-00478-y
20. Benjamini Y, Hochberg Y. Controlling the False Discovery Rate: A Practical and Powerful Approach to Multiple Testing. *Journal of the Royal Statistical Society: Series B (Methodological)*. 1995;57(1):289-300. doi:https://doi.org/10.1111/j.2517-6161.1995.tb02031.x
